# Scaffolding individuality: coordination, cooperation, collaboration and community

**DOI:** 10.1098/rstb.2021.0398

**Published:** 2023-03-13

**Authors:** James Griesemer, Ayelet Shavit

**Affiliations:** ^1^ Department of Philosophy, University of California Davis, 1 Shields Avenue, Davis, CA 95616, USA; ^2^ Department of Interdisciplinary Studies, Tel Hai College 12208, Israel; ^3^ Department of Humanities and Arts, Technion, 3200003 Israel

**Keywords:** evolutionary transition, 3Cs, MLS1, MLS2, reproducer, unit of selection

## Abstract

Processes of evolutionary transition (ET), becoming part of a new reproducing collective while losing the capacity of independent reproduction, seem difficult to track without circularity, since their features—units of selection, individuality, inheritance at multiple levels (MLS1, MLS2)—are products of one process. We describe ET in a non-circular way, noting kinds of *interactions* among community members necessary for such major transitions that are *not* instances of those same interactions within community members. Reproducing ‘systems’ tend to hybridize with environmental components, employing eco–devo scaffolding interactions forming communities. Communities are developmentally scaffolded systems of diverse members engaged in heterogeneous interactions. They may become individuals in their own right with the potential to evolve an inheritance system at the emergent community level. We argue for the explanatory benefits of treating ‘individuality’ as a special case of ‘collectivity’. We characterize an idealized sequence of collective processes—coordination, cooperation and collaboration (3Cs)—which scaffolds transitions to new forms of collective individuality: communities. Hominid evolution and learning draw attention to developmental interactions driving both dimensions of ET: new ‘levels of individuality’ and inherited ‘information systems’. Here, we outline a theoretical perspective that we suggest applies across a wide range of cases and scenarios.

This article is part of the theme issue ‘Human socio-cultural evolution in light of evolutionary transitions’.

## 1. Coordination, cooperation and collaboration: a 3Cs conceptual framework

The literature on coordination, cooperation and collaboration in humans and non-humans, their social development and their evolution, is diverse.^1^ Depending on the question of interest, any of these concepts can be treated as the general category, with the others taken as more specialized kinds or classes. We propose a particular characterization that sorts many phenomena in terms of efforts to model, track and explain: evolutionary transitions to new levels of individuality, including humans; socio-cultural problem-solving, community and group development; and the birth, death and reproduction processes of ‘individuals’ at higher levels. We view evolutionary transitions in terms of a progression of increasingly complex relations, in an idealized sequence from coordination to cooperation to collaboration (3Cs), lending a hope of understanding origins of complex, higher-level organization in terms of incremental change with each phase scaffolding the next [10].^2^ This is not a fully-fledged model but only a first and rough sketch, intended to facilitate discussion on a well-known hot topic, yet from a process-oriented research perspective. The sequence from coordination to cooperation to collaboration is idealized in the sense that it identifies one possible pathway for an evolutionary transition, analogous to ‘normal’ development as outlined in normal tables in embryology, while other pathways can thus be characterized as ‘heterochronic’ in relation to the ‘normal’ or idealized pathway. We consider it an open empirical question how (and how many) evolutionary transitions in individuality and inheritance systems proceed via this idealized path sequence.

One possible link between our idealized sequence of ‘3Cs’ and the evolutionary transition literature is that progression from coordination to cooperation to collaboration may fit ‘egalitarian’ (get together) cases better than ‘fraternal’ (stick together) cases [11,12] because, to get together, participants must first coordinate before they have the opportunity to cooperate and they must cooperate in order to find *common* ground (shared goal or purpose) upon which to collaborate. Only through collaboration can stable community (with shared identity) be established.

In fraternal cases, established networks of cooperation or even collaboration may lead to benefits (at various levels) outweighing costs of participation, so the sequence may proceed along a different pathway. For example, new academic disciplines may emerge when collaborative projects promote the creation of standard procedures that faciliate cooperation among disparate laboratories or groups and eventually lead to coordinative solutions, as changes in workflow organization give rise to segregation of jurisdictional patterns, i.e. to new specialities that coordinate their differentiated activities. In other cases, collaboration *within* small groups within a common system may create coordination or cooperation problems *among* small groups that used to see themselves in the same whole, e.g. when a cliquish group shuns interaction with other such groups and in the process impedes everyone's progress. These cases can be understood as recursive problem-solving strategies running in the opposite direction of our idealized sequence. Other pathways linking these kinds of social organization are, of course, also possible. Such pathways, beginning in ‘fraternal’ collaboration, may lead, not to further sticking-together group-individuality, but to break-up into separate groups with distinctive ‘individualities’ and no authority structure governing their interactions.

We focus on humans since they are distinctly collective, socio-cultural and descended from group-living, probably also socio-cultural, ancestral species, yet our hope is that the conceptual model applies to all life histories. Whether it makes more sense to interpret human evolution as a coming together in coordination of independent, freely evolving units or a breaking apart of already collaborating social groups will likely depend on particularities of the case at hand. We characterize the three concepts of coordination, cooperation and collaboration (3Cs) as follows [13].

*Co-ordination* is ordinating—ordering in space and time—the work of members of a collection in a place they co-occupy. This ordering may be self-organized, imposed by mutual constraints on action, or forced by agents or forces outside the interactions of participants. We envision collections as groups of individuals that may have the potential for more substantial interaction, but which interact within the grouping in minimal ways. Members of a collection do not have to work in the same space or at the same time *in order* to face coordination problems; indeed, they may be working merely to stay *out* of each other's way in space and/or time as they pursue their separate, independent projects rather than working in the same way or using the same strategy. Queues are an example of coordination in space and time in order (in part) to stay out of each other's way, as a crush of people at the front desk would not be. Of course, queues of humans (to buy tickets at a counter, for example) are orderly (when they are) owing to compliance with complex social institutions and spatial organization of human encounters, e.g. by means of ropes channelling people into single file arrangements with social conventions constraining people not to jump the ropes. Here we emphasize that queues may form through local coordinative interactions to avoid collisions when there is some kind of extrinsic spatial constraint ‘scaffolding’ the queuing, even if the constraint is not itself due to social institutions or organizations.

*Collections* may be quite ephemeral or operate over longer timescales, depending on the coordination ‘problem’ they face. People crossing the street in opposite directions form a temporary collection and co-ordinate when they each move to the right (or left) in the crosswalk, thus using the ‘same’ strategy—move right (left)—so as to achieve complementary effects to avoid head-on collision. The participants are only ‘in’ the collection (or decision/action arena) so long as they interact in street-crossing. The painted stripes on the street are an external constraint, an organizing institution and a coordinating convention. The ordination does not have to be for a shared goal or purpose other than collision avoidance, or even mutual benefit, though that may be a byproduct of the self-benefit of coordination. If there is a shared purpose, it just concerns the coordination problem in the interaction itself. This is what we mean by minimal interaction in (pure) coordination situations: cooperation extends only so far as the coordination problem at hand. Individual contributions to the collection are mainly ‘aggregative’ [14].

*Cooperation* is operating together. The operation does not have to be for a *shared* goal or purpose, or even mutual benefit, though that may be a byproduct of the self-benefit of cooperation. I may work for the paycheck. You may work for prestige. We may work for the same firm though we likely bring different experiences, skills, talents, interests, abilities and identities to our work, collected by the firm, given our different goals, but our bosses all think they are promoting teamwork merely because they call us a team. We, however, may each pursue our own goals in this group context, so long as the bosses are satisfied that we each move ‘in the same direction’. The bosses may sense a benefit to the group of our diverse contributions, but we think of those contributions as additive. We may wear the company T-shirts, but we do not feel we ‘belong to’ the group. Following Shavit & Ellison [15], we might say that our group is diverse, but not heterogeneous: as individuals, we vary one to the next, but our interactions are limited to each contributing to the group ‘effort’ such that the group ‘effect’ is (to us) a byproduct of our individually motivated contributions. Our contributions to effects on the group are interactive and non-aggregative [14], but only in limited ways. We ‘enter’ the same space (or decision arena) *in order to* cooperate, rather than merely incidentally, as with coordination processes, likely because our individual goals require a group context to achieve them. I do not get the paycheque if the firm does not make a profit by turning out the widgets. You do not get the prestige if the firm limps along rather than rises to prominence in the business world by producing desirable widgets. We may cooperate to each get what we want out of working together rather than separately. Cooperation involves coordinating so as to get what you want/need out of working *together*, possibly for outcomes not achievable in isolation. Nevertheless, it always makes sense to calculate or measure effects of cooperation in terms of the marginal benefits to individuals, because the currency of cooperative interaction is the same as for individual action. Lions may need to cooperate in hunting to bring down a 900 kg antelope, but it makes sense to calculate the benefit per lion in weight of meat consumed by each relative to the energetic cost each endures in their contribution to the group hunting effort. Cooperation may have not only direct effects on group traits and structure, but also indirect effects on individuals that lead in turn to feedback on groups, e.g. through choices to leave or join subgroups, engage or not engage in interactions within the group, through work that changes what particular subgroups stand for or do, or which change the operation or character of the group—its ‘identity’.

*Collaboration* is labouring together. The labour is for a *shared goal* (including unrealized or unfulfilled goals); otherwise it is merely cooperation, which could be working for separate purposes or even at odds, but in a place entered for that goal, purpose or reason. When such labour is in the context of ‘community’ (see below), it may contribute to a ‘sense of belonging’ or group identity, which might be interpreted as trust in the reliability of the group context to deliver both individual and group benefits, as an additional aspect of evolutionary transition to group-level individuality or as a feature of community maintenance in already established communities. Reliability is thus an emergent community property. The heterogeneous interactions among diverse group members contribute in substantially non-aggregative ways to group benefits such as group-reliability that cannot even be *recognized* as group benefits without reference to the special interactions engenderged by the group. It makes little or no sense to calculate marginal benefits distributed among members. When a sports team wins a game, it is purely derivative to say that the team members each ‘won’ the game. We might say that these group benefits are assignable to ‘emergent’ group properties (e.g. the *team's* performance in the game) and not assignable to contributing group members (except in the sense that calculations of marginal benefits are always possible, even if effectively meaningless).^3^

The *evolutionary* question is how collaboration might emerge out of cooperation and cooperation out of coordination. Specifically, the socio-cultural evolution of human convention-driven social institutions, with group structures stabilized more or less to form ‘communities’, is the focus of this paper. Here, the phenomenon of interest is mechanisms joining and holding disparate cooperative trait groups (reference groups, sub-communities) together in a bundle, operating long enough and coherently enough to manifest a degree of individuality at the group level in respects shaped by the intersecting reference groups, e.g. individuality in the sense of autonomous entities defined by their ‘organizational closure’ [16].

Working *for* a shared goal, purpose, group property or outcome in a community, rather than just working *in* a connected collection, is a characteristic problem of contemporary social organization.^4^ Community pursuit of shared goals in collaborations is not merely the pursuit of cooperative outcomes through individual interactions in a connected network of interacting individuals—referred to as a ‘conexus’ from here on. Community involves further features of interacting networks of participants beyond cooperative interactions. Following Shavit & Ellison [15], we emphasize heterogeneity in addition to diversity, that is, we look not just at the relative abundance of different members and groups (diversity) but also at the different interactions and structural positions these members and groups hold in social networks (heterogeneity). Those further features may qualify the change from coooperative conexuses to collaborative communities as ‘transitions’ in the sense that theorists of evolutionary transitions in individuality and inheritance systems address for evolving systems.

Whether the relevant sort of further evolution, called the ‘transformation’ phase of a transition process [12], toward new transitions is occurring among humans now (e.g. an artificial intelligence (AI)–human transition, see [17–19]) is an empirical question of great complexity. It is not yet clear how to think about it, much less empirically evaluate it. It seems clear that additional conditions beyond merely displaying ‘Darwinian’ properties (heritable variation in fitness) are required [20,21]. We suspect that a key element of such transformations will be what we call below a ‘sense of belonging’, ‘trust relations’ or ‘identity’ on the part of humans involved in social communities of humans and technologies such that humans find ‘community’ with AI and not only with other humans.^5^

Processes of evolutionary transition by human individuals and organizations might be conceptually modelled in terms of orderings of processes of coordination, cooperation and collaboration into stages, so long as we distinguish the idealized historical order of processes in evolutionary transition (coordination to cooperation to collaboration) from a developmental order of problem-solving in human socio-cultural systems, e.g. from recursive collaboration problems to cooperation problems to coordination problems. In addition, other developmental orderings may recurse in other directions among the three, depending on context, circumstance and system organization, than those bringing about a socio-cultural human transition to community-grade social organization.

## 2. 3Cs conceptual model

The structure of concepts (table 1) in the 3Cs model presents a common framework for incorporating socio-cultural as well as bio-social development in models of evolutionary transition. In this section, we explore the practical meaning of the 3Cs, mark their added theoretical relevance via analogies and disanalogies with other existing useful models, and briefly illustrate their explanatory power for other ETI case studies (independently studied in detail across this special issue).

**Table 1.** 3Cs (coordination, cooperation, collaboration) ordered in stages of evolutionary transition.

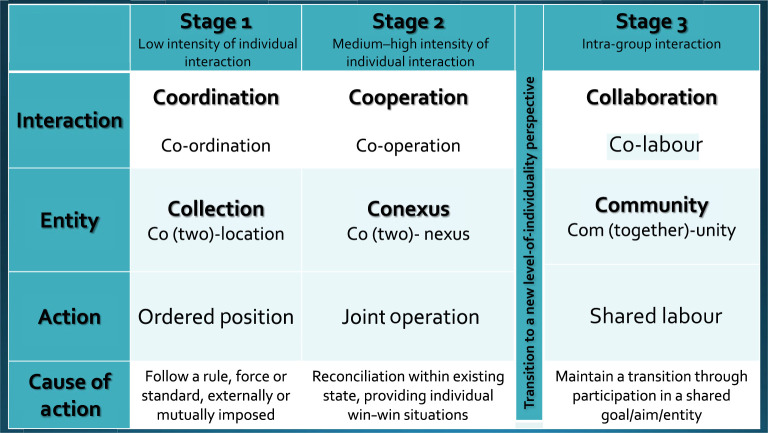

The 3Cs involve three kinds of interactions, ideally sequenced as stages in an ETI process and linked to three levels of unithood or phases of ‘individuality’: collection, conexus and community. Each new level of unithood further increases density and complexity of interaction structure, from coordinative to cooperative to collaborative interactions. The last—collaborative interactions within a community—involve a shared-construction of interactions [23] that marks a transition in individuality and potentially a new inheritance system (see below).

To visualize this model, ‘collection’ brings individuals into pairwise interactions that constitute collections of binary interactions through which coordination problems can be solved. ‘Conexus’, i.e. connected collection, brings individuals into systems of binary interactions such that the whole system of interactions constitutes a web of junctions and meeting points. ‘Community’ organizes individuals in intersecting reference groups into a rich network of heterogeneous multi-way interactions where some interaction subsets have become entrenched so that most other interactions depend on them for their operation and existence [10]. These entrenchment relations knit the interaction system and reference groups into (more or less) dynamically stable organizations that have many of the features of group-level individuality.

There is a rough positive analogy between our sense of ‘group structures’ in these three levels of unithood (collection, conexus and community) and the contrasts drawn in the literature between individual selection, multi-level selection 1 (MLS1, group effects on individual fitness) and multi-level selection 2 (MLS2, group effects on group fitness) [24–26]. But rather than *contrast* MLS1 with MLS2, i.e. where only individuals or also groups are the reproductive entities in the system, we see *progressions* or developmental capacities for change from one dynamical regime to another. This change may foster evolutionary transitions from potentially unstable MLS1 situations into more stable MLS2 situations with the emergence of new units of individuality [25,27]. In the Neolithic Revolution in the southern Levant, an example we will later return to, egalitarian hunter–gatherer site-specific cultures became hierarchically structured agricultural communities on a short timescale, yet without any scientific agreement how to weigh the evidence of climatic change, intra- or inter-group conflict and often without even a change of locality [28]. Whether the emergent new units are more than just ‘trait groups’ that form ephemerally (e.g. beetles forming a group owing to their common individual behaviour of foraging on leaves) and then dissolve prior to panmictic mating [29] or include some capacity for community-level ‘demic’ reproduction is an empirical question about the post-transition evolution of the population-level network-interaction structure of such communities (see below).

Thus, our evolutionary transition question sits between those of scholars who aim to focus on the evolution of individuality *per se* [11,30] and those who retain the second meaning of evolutionary transitions in inheritance [12]. Our question regarding human social transition is whether transitions are only in individuality or also involve transitions in inheritance systems manifested in higher levels or more complex organizations of reproduction, whether the latter involves fully emergent individuals or not [12]. In our framework of concepts, answers to our evolutionary transition question for particular cases and situations also turn on the empirical determination of whether those particular systems form trait group or demic communities, since the latter, but not the former, are candidates for higher-level reproducers [31] that may, post-transition, evolve the developmental integration necessary to count them higher-level individuals.

One disanalogy between the 3Cs perspective of evolutionary transition in individuality (ETI) and the original context of MLS1 versus MLS2 as a means of addressing the debates over group selection is that the 3Cs are not necessarily about levels of *selection.* Rather, they are about levels of individuality and reproduction, which have a complicated relationship to levels of selection [32]. We characterize levels of collectivity in terms of properties of density, intensity, higher-level emergent ‘jointness’ and heterogeneity, which relate to properties of complexity and connectivity, but also to interaction strengths and symmetry or directionality.

3Cs collectives involve (perhaps temporary) interactions through which individuals coordinate because they happen to be in the same place (spatio-temporal co-location) and thus must interact in order to continue to pursue whatever process trajectories they were individually following that led to the interaction. This spatio-temporal contiguity in turn may have been forced on participants either by exogenous environmental circumstances or by conexuses or communities. In these cases, the traditional view is that ‘groups’ are too ephemeral and the interactions too weak (or not of the right sort or number) to form *collective* reproductive units. In MLS1, groups of individuals organized so as to cooperate for individual benefit (‘conexuses’ in 3Cs) form dense interactions that are somewhat stabilized, but the persistence of individuals in various ‘reference’, ‘trait’ or ‘task’ groups operates at a potentially unstable, dynamic equilibrium with respect to the traits and processes forming the conexus.

Bourrat [21], following Black *et al*. [33], offers a model for the evolution of Darwinian properties at the level of collectives. The main conceptual point is to distinguish Darwinian individuality from Darwinian-*like* individuality. In the former, following Clarke [34] a collective exhibits individuality if it has the Darwinian properties of heritable variation in fitness plus some internal individualizing mechanism (policing + demarcating mechanisms). The individualizing mechanisms are internal in the sense that they are due to member ‘particles’ of the collective and confer *resilience* in the face of environmental perturbation. Bourrat considers models in which an ETI is initiated by ecological ‘scaffolding’ features of the environment rather than by internal (e.g. genetic epistasis or pleiotropy) individualizing mechanisms. His models suggest that to get to genuine Darwinian individuality, collectives must ‘endogenize’ (i.e. bring inside the individual) their scaffolded features so that they achieve this resiliency to environmental perturbation without ‘outside’ help. Otherwise, undoing or releasing the system from scaffolding results in the ‘de-Darwinizing’ of the collective, just as the scaffolded transition to collective individuality had ‘de-Darwinized’ the component particles [20] and effected a ‘transfer’ of fitness from particle to collective level [30]. According to Bourrat, real individuals are not ephemeral in this way. We applaud Bourrat's (and Black *et al*.'s) recognition in these models of the role of scaffolding, as well as his view—in light of the possibility of niche construction of scaffolding—that internal and external causes and mechanisms need not be mutually exclusive. However, we think his reliance on classical island migration models to illustrate a scaffolded imposition of a linkage between cell growth rate and dispersion in the models may neglect the modelling tradition associated with Wright's shifting balance process [26,35].

Our project seeks to understand a variety of forms of *heterogeneous* collectives whose diverse members contribute to a network of interactions, such that costs and benefits of joining or leaving a group are dependent on the mechanistic *interaction structures* of the groups and not merely on fitness consequences at either level. Marginal fitness calculations will be insufficient measures in such contexts. Differently put: we suggest an eco–devo approach to evolutionary transitions to ‘individuality’ that is more pluralistic than classical Darwinian individuality approaches allow, with different kinds of individuality beyond the ‘Darwinian’ theme explored by philosophers of biology such as Godfrey-Smith, Clarke or Bourrat. Our view is that *community* can be seen as a different *kind* not of individuality, which is not only an issue of Darwinian properties at collective level, even if supplemented by individualizing mechanisms (whether externally scaffolded or internal) that regulate growth rate and dispersion, as Black *et al.* have modelled [33].

While Bourrat [21] has already identified one missing ingredient from classical discussions of evolutionary transitions in Darwinian individuality in his emphasis on *resilience* to environmental perturbation, we propose another missing ingredient linked to our idea of associating collaboration with community: the *reliability* of the community to provide the social context in which resilience in Bourrat's sense can be secured *at the community level*, i.e. where ‘endogenization’ moves scaffolding effects into the *community* rather than into a lower-level individual. We hope to explore such models in future work.

A key point of our approach is to mobilize concepts so as to recognize *two* kinds of unstable dynamic equilibria occurring in knotted conexus interactions in connected collections. First, individuals can *move* (migrate) among ‘reference’ groups such that when an individual moves, the pattern, intensity and density of interactions distributed within and among reference groups can shift in ways that affect group ‘traits’, including membership conditions. Second, the reference groups themselves can move their trait boundaries (e.g. religious groups might shift doctrine, banks might become investment houses, progressive political parties might moderate or moderate ones become radical) such that the reference groups to which individuals belong in various combinations can shift (e.g. a person can no longer ‘recognize’ their party owing to political radicalization in the latter, though they might continue to stay a voting member in that party owing to another, social rather than ideological, dimension of belonging). In a nutshell, groups can move away (in trait space) from relatively stable individuals, just as individuals can join or leave relatively stable groups. Wimsatt & Griesemer [36] used such a reference group approach to analyse cultural development and evolution.

This duality of kinds of instability can be understood in terms of how David Sloan Wilson's original trait group models were set up in contrast to interdemic selection models [29]. In Wilson's trait group models, organisms mate at random across a meta-population of trait groups, but experience selection (both individual and MLS1-group selection) within ‘their’ trait group. In interdemic selection models, organisms mate at random within their deme and experience individual and MLS1-group selection within their deme, but demes also reproduce (see below) in virtue of organisms migrating among demes or via deme extinction and recolonization in accordance with Wright's shifting balance theory of evolution ([37,38]; see also [26,35,39,40]). Thus, whenever there is variation among sub-populations with effects on fitness *at that level*, there is an opportunity for MLS2-group selection [26]. In terms of the 3Cs characterization of levels of social groups (collections, conexuses and communities), members of a group can migrate among such groups *within* organism and group generations (i.e. in the period when individual and MLS1-trait group selection occur), rather than assign all migratory or movement behaviour *between* trait group or demic generations as classical models assumed.

Thus, where classical group selectionists imagined a fixed, developmentally stable group composition in the ‘selection’ phase of a group life cycle alternating between bouts of selection and reproduction, we imagine degrees of stability of trait groups themselves such that they can change in character, i.e. ‘develop’, *during* the course of their trait and demic group interactions with their environments. This means that selection and reproduction are more or less intertwined over the development of the cycle, rather than isolated into distinct phases. This also means that development (at the group level) as well as group selection can alter the reproductive and evolutionary dynamics of group-level phenomena. It also supports a view of the stages of evolutionary transition in which transition to a new level of reproduction may indicate the emergence of a new level of individuality, but not yet of a transition in inheritance system. The latter may require further adaptive evolution of the developmental organization of the emergent-level reproducers ([31,41,42], also [32]), which may also involve the further jointness of the individuals at the new level (see below).

The twin effects of these two forms of dynamic (in)stability suggest that it is likely to be very difficult to predict whether a particular conexus will evolve to a point where transition to community organization is plausible, under even specified conditions, though Bourrat's models hint at some timescale conditions relating growth rate and dispersal [21]. Specific conditions can be conceived to produce roughly three categories of outcome. First, Darwinian-like groups [21] can dissolve (as classical trait groups do) into merely individual coordinative regimes of individual action if individuals jettison affiliation with some of their reference groups. The human phenomenon of ‘choosing a camp’, illustrates this point. When individuals identify with/against (join/leave) a singular reference group, e.g. political or religious affiliation, they make a range of intense commitments to coordinative and cooperative action. Second, groups can persist (on some timescale) as a potentially unstable conexus, maintained by external ecological scaffolding and by the internal dense coordinative interactions of those individuals at the centre of the interaction network (i.e. those with high connectivity). Intersections in a conexus (i.e. strongly connected reference groups cross-linked by one or a few individuals belonging to more than one reference group at a time) may hold the collective together despite the component subnetworks being organized around ephemeral coordinations only. Third, groups can stabilize the network of interactions within and among intersecting and non-intersecting reference groups, making their stability a matter of self-organizing processes that integrate several to many conexuses into a community. For example, a reoccurring behaviour can be stabilized into a stratified ‘social role’ or ‘line of work’, as in the transition from hunter–gatherer sites to agricultural communities.

In the last case, stabilization of the network affords the opportunity for evolution to entrench particular patterns of interactions that may be heterogeneous across subgroups, e.g. resulting from combining one sort of interaction within a political reference group with another sort of interaction within a religious group into a politico-religious community. When individuals stay in their combinations of intersecting reference groups, and group boundaries stay sufficiently fixed, *other* interactions among individuals not in reference group intersections can form; the latter interactions can be stably assembled on top of—i.e. as depending on—what become ‘core’ interactions. As social structure evolves from coordinative to cooperative to collaborative, core interactions become generatively entrenched. This is a crucial point in an ETI process. A generatively entrenched interaction is a core interaction that later-evolved interactions will depend on for their existence and character, so that perturbations of it will tend to be catastrophic to the whole system while changes in the later interactions tend to lead to tolerable system changes [14].

This process of generative entrenchment drives the transition from conexuses to communities. When systems running on cooperation evolve into entrenched systems of collaboration, these in turn may, over time, incorporate inter-actions among disparate collective conexuses as ‘intra-actions’ [43] within an expanded, stabilized community. It is important, however, to notice that transitions from conexus to community are highly contingent, even if contingently *irreversible* [44]. There is nothing inevitable about the evolution of collaboration or transition to a new level of individuality, yet once this process occurs the new level of individuality and inheritance system is unlikely to return to the previous state, unlike many other evolutionary results. In the cases of human evolutionary transition of interest here, if there is a transition, it is marked by a change in the way (human) communication is organized (and propagated), not only a change in the way genetic information, or even group size, is transmitted.

## 3. Group-level reproduction

Caporael [45] argues that cognition is truly social when there is cognition about the group itself, cognition specialized for group living, rather than (we might add) merely concerning a member's role or place or projects carried out in group context. More colourfully, cognition cannot just be ‘in the head’ of individual cognizers because they are parts of complex groups to which individual cognizers have incomplete, imperfect access. This is the situation we describe as ‘community.’ It follows from Caporael's concept of a social group (see Caporael *et al.* [46]) that for a community to be reproduced, it must be exported as an organized generative package larger than a single individual member. Community reproduction depends on more than the diversity of members: the heterogeneous structure and dynamics of their interactions need to be propagated in order to ‘transmit’ organization. Such generative organization is required because no one individual migrant can carry even a symbolic representation of the whole community structure in their head, let alone sufficient knowledge of each differentiated role of diverse members specializing in divided labour, who interact in heterogeneous ways, including some roles no single member will likely have experience in or even be aware of.

In this section, we conceptually model the reproduction of groups, e.g. social groups making more social groups, with the potential for heritability relations between ancestral and descendant groups. For clarification, an analogy with popuation genetics will be used, utilizing approaches to modelling inheritance and evolution in subdivided biological populations undergoing a shifting balance process. Inheritance processes, *sensu* Griesemer [41], involve propagation from parents in the production of offspring, through a process of sampling their material parts to produce small generative groups of parts (propagules). A gamete or spore is a one-cell ‘sample’ of parental material. A small group of migrant individuals is a ‘sample’ of a deme or population. These are of course not typically *random* sampling processes, but it helps to see some commonalities among these processes to view them as sampling processes.

On Griesemer's account, in what are called inheritance systems, this kind of sampling process is evolved to a degree that ensures the propagule reliably develops into an offspring that can participate in continuing a lineage. Reliability can involve a trade-off between developmental resources carried within and ecological scaffolding outside the propagule. This sampling need not propagate genes or genomes. On the other hand, sampling propagates more than mere resemblance in virtue of the offspring propagule having originated from parts of the parents. Inheritance systems are *evolved* to propagate sufficient organization of material parts so that a propagule can develop into a similarly organized reproductive entity [41]. The propagule, in other words, carries developmental capacities from one generation to the next and this is the mechanistic basis of heritability relations. The value and advantage of an evolved inheritance system over just haphazard ‘similarity’ due to sharing of material parts is the enhanced respects and increased degree to which that generative developmental *organization* persists through reproduction, so that the offspring can reliably ‘repeat the assembly’ [47] of a functioning resilient organism, as did its parent(s).

Abstracting further, when members of a group leave to form new groups (either by founding new groups or by joining existing groups), they sample the social-behavioural and phenotypic as well as genetic organization of the group they came from and to some degree, in some respects, propagate the parental organization to the new population, carrying in those respects and degrees the parent's capacity to develop (including capacities to engage scaffolding). We characterized this organization in terms of diversity of nodes and heterogeneity of interactions and structure in a social network. More generally, our interest is in forms of developmental organization that may be carried to offspring that ‘sample’ parental organization.

Returning to the topic of human social groups organized in collaborative communities, we can think of the problem of community inheritance as the reproduction of developmental organization: migrants who sample the diversity of nodes and heterogeneity of interactions and interaction of a community network to repeat the assembly of community-grade network organization through a process of development. On the other hand, human social group migration can surely also be a means for the products of evolution to modify the mechanisms of the evolutionary process that created them. Moreover, recent work shows how learning theory might lead the way to evolutionary models: Watson & Szathmáry argue that selection at one level of organization can operate like unsupervised learning at a higher level of organization [48].

The general question for evolutionary transition in inheritance systems for humans is: What is required for societies organized in communities to carry developmental capacities sufficient to regenerate (repeat the assembly of) a lineage-continuing social organization of diverse members with heterogeneous interactions, like its parent societies? Answering that question, for specified conditions of group-level evolution, will characterize not only the conditions for the emergence of a new, community-level individuality of groups, but also the opportunity for a new, community-level inheritance system, i.e. both aspects of an ETI.

Processes driving the integration of conexus cooperative networks may change them. Super-networks of interconnected sub-networks may transform into systems where sustaining the heterogeneity of interactions among diverse nodes of a joined system becomes a focal goal of every participating member. Integration means that removal of node or interaction types disrupts social organization in such a way that nascent or emerging trust relations that help organize community fails to serve as a focal goal. Collaboration retreats to conexus level or falls apart entirely into cooperative interactions. Put differently, community requires ‘organizational closure’ [16] of the system of social interactions to achieve and sustain community-grade organization. Hence, community constitutes a new level of individuality.

At the same time, arrival at this new grade or level of organization also affords the possibility of community-level reproduction by means of samples of community migrating to found new groups or merge (fuse) with other existing groups. If the new groups further evolve for transitions from coordinative to cooperative to collaborative organization, they may be organized such that to export their organization to found new collectives, conexuses or communities, sampling must be done in a way that carries the relevant developmental capacities.

A simple thought experiment drawing on Shavit & Sharon's account [28] of the evolution of Neolithic human societies illustrates these points. Suppose a society is organized into diverse members with a heterogeneous division of labour among hunters, gatherers, farmers and shamans. Suppose further there are heterogeneous interactions among types of ‘occupation’ (and probably within types as well). For example, members within types will typically have to organize into task groups of hunters, or gatherers, or farmers, or shamans to accomplish their type-specific cooperative or collaborative purposes if the site-specific culture is of a size such that no one member can efficiently perform all of the differentiated roles within a task. Then sampling such an organization might require smaller migrant groups to include one or more members of each type, and include sufficient heterogeneity of ongoing interaction types among group members, if that cultural organization is to be successfully assembled at a new site. In addition, migrants may require special group-specific properties: farmers capable of fencing as well as tilling, hunters capable of surveilling novel terrain or innovating tools (e.g. for fishing rather than spear hunting), gatherers capable of recognizing new edible seed plants, and shamans capable of leading the setting up of a ‘temple’ rather than only performing rituals.

Evolutionary innovation in sampling for reproduction of developmental organization is characteristic of any inheritance system. On this first rough sketch of a conceptual model of 3Cs, inheritance systems at new levels seem likely to evolve in tandem with evolutionary transitions to new levels of individuality in so far as the latter involve diverse members with heterogeneous interactions because basic processes of group formation, dissolution, joining and leaving require the propagation of developmentally stabilized order. Unlike those who think distinguishing evolutionary transitions in individuality from evolution of inheritance systems simplifies the explanatory project and distinguishes different processes, we think that the two go hand in hand and that satisfactory models of empirical cases of evolutionary transitions will likely be better understood in terms of the 3Cs: transitions from coordination in collectives, to cooperation in conexuses, to collaboration in communities. This three-stage model of evolutionary transition in terms of 3Cs suggests that Maynard Smith & Szathmáry's distinction of ‘limited’ and ‘unlimited’ [44] might be further characterized in terms of the specific features of mechanisms yielding coordination, cooperation or collaboration.

## Data Availability

No data were reported in this study.

## Endnotes

1 Organizational research in management studies focused on teamwork examines all three concepts within and among business organizations [1]. Evolutionary anthropologists study the evolution of cooperation in humans [2], and evolutionary biologists since Darwin study the evolution of cooperation in non-human and human organisms [3–7]. A reviewer pointed out a range of definitions of cooperation in evolutionary biology from ‘any adaptation that has evolved, at least in part, to increase the reproductive success of the actor's social partners’ [8] to ‘social interactions characterized by costs to an actor and benefits to other conspecifics’ [9], and in social psychology as ‘those social interactions in which actors incur in some costs to altruistically provide benefits to a partner’ (quoting the reviewer). While we agree that cooperation as understood in these fields usually involves a cost–benefit interaction, and thus, at least in that respect, the definitions seem similar to one another, notice that in the first case (adaptation), cooperation is defined by an outcome or product, in the second case by a condition (on a process or product), and in the third case by a process but with conditions on the outcome. Indeed, while these definitions may seem similar, they are incompatible, since the first rules out by definition non-adaptive cooperation while the second only permits cost–benefit conditions of altruism among conspecifics, and the third permits similar conditions to ‘partners’. We address these differences in the text to position our conceptual project in relation to these others.

2 The common meaning of scaffolding refers to a temporary *structure* or an *interaction framework* that supports or facilitates a process or action, e.g. in the poles and platforms of a builder's scaffold or the caregiver or teacher who facilitates learning in a child or student. Caporael *et al.* [10] extended this meaning to processes of biological development, evolution and cognition.

3 Academics have a familiar experience of this phenomenon. Most research collaborations are hardly assignable to individuals, yet journals and universities routinely demand allocations of credit, either in terms of quantity of effort contributed, or in terms of specific assignable tasks (‘conceived the project’, ‘wrote the grant’, ‘collected the data’, ‘analysed the data’, ‘drafted the manuscript’)—as though each of the tasks of conceiving, writing, collecting, analysing or drafting research can be assigned to individuals, *as if* without the product being wholly the result of interactions among participants.

4 Nor is adjustment of individual properties or interactions merely due to operation in a collection.

5 This may offer a way of interpreting philosopher David Chalmers' recent embrace of ‘virtual realities’ as genuine reality of sociality facilitating collaboration, cooperation and coordination in virtual worlds organized by digital rather than physical–social technologies, infrastructures and socio-cultural contexts [22].
